# Multiple Output Gaussian Process Model for Predicting Low Birth Weight in Medellín, Colombia: An Alternative to Conventional Machine Learning Models

**DOI:** 10.1007/s10995-026-04284-x

**Published:** 2026-06-29

**Authors:** Diego Alejandro Salazar Blandon, Hernán Felipe García Arias, Juan José Giraldo Gutiérrez

**Affiliations:** 1 School of Nursing, University of Antioquia, Medellin, Colombia; 2 SISTEMIC research group, University of Antioquia, Medellin, Colombia; 3 National Heart and Lung Institute, Imperial College London, London, UK

**Keywords:** Low birth weight, Machine learning, Models

## Abstract

**Objective:**

To evaluate the methodological feasibility of a heterogeneous multi-output Gaussian process model for jointly handling a continuous birth outcome and its clinically used binary representation in routinely collected perinatal data, and to compare its predictive performance with that of conventional single-output models.

**Methods:**

Routinely collected live-birth certificate data from Medellín, Colombia, covering births from 2012 to 2021, were analyzed. After cleaning and class balancing, the analytic dataset included 32,110 records. A heterogeneous multi-output Gaussian process model was trained to jointly model birth weight in grams with a Gaussian likelihood and low birth weight status with a Bernoulli likelihood. Predictive performance was compared with that of conventional single-output regression and classification models.

**Results:**

The heterogeneous multi-output Gaussian process model achieved acceptable predictive performance (R² = 0.67 for birth weight and accuracy = 0.845 for low birth weight classification), with results comparable to those of models fitted separately for each task. These findings support the practical feasibility of modeling heterogeneous outputs within a single probabilistic framework.

**Conclusions:**

In this application, the heterogeneous multi-output Gaussian process model was a viable methodological alternative for jointly modeling birth weight in grams and its binary low-birth-weight classification. This study should be interpreted primarily as a methodological demonstration of a flexible multi-output framework in perinatal data that may be extended in future studies to jointly model other outcomes of greater direct relevance to public health.

**Supplementary Information:**

The online version contains supplementary material available at 10.1007/s10995-026-04284-x.

## Significance

What is already known on this subject?

What this study adds?

Low birth weight is an important indicator of neonatal risk and remains a relevant target for prediction in maternal and child health research. In this study, its importance lies in serving as a clinically interpretable example for evaluating a flexible multi-output framework capable of jointly modeling a continuous perinatal measure and its binary clinical representation.

This study shows that a heterogeneous multi-output Gaussian process model can jointly model a continuous perinatal outcome, birth weight in grams, and its related binary clinical classification, low birth weight status, within a single probabilistic framework. The model achieved predictive performance comparable to conventional single-output approaches, supporting the methodological feasibility of jointly modeling related continuous and dichotomous outcomes in routinely collected perinatal data.

**Supplementary Information:**

The online version contains supplementary material available at 10.1007/s10995-026-04284-x.

## Introduction

The World Health Organization (WHO) defines Low Birth Weight (LBW) as a birth weight of less than 2,500 g, regardless of gestational age or the reason for low weight. This definition is based on epidemiological observations indicating that children weighing less than 2,500 g are approximately 20 times more likely to die than heavier babies (Blencowe et al., 2019). LBW is a leading cause of neonatal mortality and is associated with medium and long-term medical problems. Children with low birth weight have a higher incidence of neurological deficits, growth alterations, cognitive deficiencies, and non-communicable chronic diseases. Evidence has shown that LBW is related to sociodemographic factors associated with parents (Benjumea et al., 2009), the number of prenatal visits, among others. Therefore, it is considered an important public health indicator for maternal and newborn care, due to its link with living conditions, population health, and medical care for pregnant women. According to official figures, this health indicator has shown an upward trend in recent years in Colombia (DANE, 2024). Thus, having a model that anticipates LBW by identifying modifiable risk factors could reduce infant mortality rates and improve health indicators.

A growing body of research has investigated the prediction of LBW using both conventional statistical models and, more recently, machine learning approaches. This literature spans a wide range of predictors, modeling strategies, and clinical settings. In a systematic review of prognostic prediction models for adverse birth outcomes, Muche et al. (2024) identified 115 models, including 17 specifically developed for LBW, and found substantial heterogeneity across studies, with limited external validation. Illustrative examples include a large multicountry prospective cohort based on routinely collected prenatal variables (Patterson et al., 2023), a pragmatic maternal risk score developed in Ethiopia (Hassen et al., 2020), and a comparative study showing the added value of combining clinical, biometric, and Doppler information in high-risk pregnancies (Sanchez-Martinez et al., 2024). Reviews of artificial intelligence in perinatal health have also documented increasing use of machine learning for outcomes such as preterm birth, birthweight, hypertensive disorders, and perinatal mortality (Ramakrishnan et al., 2021). Nevertheless, the field remains constrained by methodological heterogeneity, incomplete reporting of calibration, and the scarcity of robust external validation. Against this background, the present study examines whether a multi-output strategy that jointly models birth weight and its classification as LBW may offer added value over conventional single-output approaches.

Most conventional supervised learning methods are designed to predict a single outcome at a time, whether continuous or categorical, and are therefore commonly described as single-output or single-task approaches. However, many real-world problems involve multiple related outcomes, which has motivated the development of multi-output models. Among these, Multiple Output Gaussian Processes (MOGP) provide a flexible probabilistic framework in which dependencies among outputs can be represented through shared latent structure (Liu et al., 2018). According to Xu et al. (2020), although traditional single-output supervised learning remains widely used and often performs well in practice, multi-output learning offers an attractive alternative when outcomes are related and may benefit from joint modeling.

The Heterogeneous Multi Output Gaussian Process (HETMOGP) model proposed by Moreno et al. (2018) is an extension of the MOGP framework in which the target variables may be of different natures, allowing continuous, binary, or categorical outputs to be modeled jointly. In these models, each output is associated with its own likelihood function, whose parameters are represented through latent functions generated from Gaussian processes. More recently, Giraldo et al. (2022) proposed an extension of this framework in which the likelihood parameters are generated through convolution processes rather than through a simple linear combination of GPs.

This study aimed to train and evaluate an HETMOGP model, using the implementation by Giraldo et al. (2022), to jointly model two related outputs derived from the same birth outcome: birth weight in grams (continuous) and low birth weight status (binary, defined using the conventional 2,500 g threshold). Rather than treating these outputs as clinically independent endpoints, the study assessed whether a heterogeneous multi-output framework could simultaneously accommodate a continuous perinatal measure and its standard clinical categorization in real-world public health data. Predictive performance was compared with that of traditional single-output approaches commonly used in health research, including linear regression, logistic regression, support vector machines, and decision trees. Accordingly, the primary contribution of this study is methodological rather than etiologic or clinical.

## Methodology

The data were published in MeData (Alcaldía de Medellín, 2019) an institutional repository of the city of Medellín that aims to promote the appropriation, openness, and use of data as a tool for governance, citizen action, and decision-making, under a Creative Commons license. This dataset contains information related to births in the Municipality of Medellín between 2012 and 2021, comprising 272,526 records and 40 variables.

After an initial selection of characteristics based on clinical relevance and utility for the model, and an initial data cleaning process—removing incomplete, duplicate, and outlier records—a dataset with 20 characteristics and 146,801 records was obtained. Among these records, 10.9% (16,055 births) had low birth weight, indicating a significant imbalance in the variable of interest. To address this, random sampling was performed on the category without low birth weight to create a balanced dataset of 32,110 records and 22 variables, which are described in Table 1.

**Table 1:** Variables Included in the Analysis

Name	Description
AREANAC	Area of Birth
SIT_PARTO	Place of Delivery
SEXO	Sex of the Live Birth
MES	Month of Occurrence
ATEN_PAR	Attending Personnel
T_GES	Gestation Period
NUMCONSUL	Number of Prenatal Visits
TIPO_PARTO	Type of Delivery
MUL_PARTO	Pregnancy Multiplicity
IDHEMOCLAS	Blood Type of the Newborn: Blood Group
IDFACTORRH	Blood Type of the Newborn: RH Factor
EDAD_MADRE	Mother’s Age at Delivery
EST_CIVM	Mother’s Marital Status
NIV_EDUM	Mother’s Education Level
N_HIJOSV	Number of Live Births
N_EMB	Total Number of Pregnancies
SEG_SOCIAL	Mother’s Health Insurance
EDAD_PADRE	Father’s Age at Delivery
NIV_EDUP	Father’s Education Level
COMUNA_RES	Mother’s Residence
**PESO_NAC**	Birth Weight
**BAJO_PESO**	Low Birth Weight Classification

Before training the models, a one-hot encoding transformation was applied to create dummy variables reflecting categorical variability numerically, using zeros and ones. For the HETMOGP model, due to the complexity of optimizing the loss function, Label Encoding (assigning a numerical value to each category of the variable) was used to avoid increasing the problem’s dimensionality. Numerical features were transformed using the MinMaxScaler() method to achieve balance in the scales of the variables. Specifically, variables such as age and number of prenatal visits are expected to have similar scales after transformation. Additional details on the analytic dataset, preprocessing steps, and variable coding are provided in the Supplementary Methods (Sections S1–S2).

*Ethical considerations* This study used retrospectively collected, anonymized secondary data obtained from the MeData repository (Alcaldía de Medellín, 2019), which provides open-access records under a Creative Commons license. No direct personal identifiers were available to the investigators, and all analyses were conducted on de-identified records at minimal risk to participants.

### General Approaches Based on Gaussian Processes with Multiple Outputs

In this study, the multi-output setting was defined by pairing a continuous outcome (birth weight in grams) with its binary clinical representation (low birth weight: yes/no). This choice was made to evaluate the feasibility of a heterogeneous probabilistic model under a simple and clinically interpretable output structure, in which the model must accommodate different likelihoods within a single framework. The binary output was not assumed to contribute independent biological information beyond the continuous measurement; rather, this application was used as a proof-of-concept for handling heterogeneous outputs in routinely collected maternal and newborn data.

For this approach, due to the computational complexity in optimizing the loss function, a feature importance analysis was performed prior to training using Light Gradient Boosting Machines (LGBM), which is ideal for large datasets with a mix of quantitative and categorical variables (Ke et al., 2017). Additional implementation details for feature selection and HETMOGP specification are provided in the Supplementary Methods (Section S5).

### Baseline

First, feature selection was performed using a binary prediction approach, from which the most relevant features were chosen to achieve a cumulative importance of at least 80%. Next, an initial experiment was trained and validated with an initial parameter configuration. The HETMOGP model is assumed to have two outputs: one output to account for the continuous variable PESO_NAC (in grams) modeled using a heteroskedastic Gaussian likelihood, which, according to Giraldo et al. (2022) allows for flexible modeling of continuous outputs (as in regression). A second output to account for the binary variable BAJO_PESO (Yes/No or 1/0) modeled using a Bernoulli likelihood. Additionally, based on initial parameter tuning tests and execution times, the Adam optimizer (Adaptive Moment Estimation) and the VO optimizer (Variational Optimization) was selected for its benefits in rapid convergence. Finally, some configuration parameters to train the model were set as follows: 250 instances per batch (Minibatch), 50 inducing points which allow estimating the posterior from informative latent variables (Inducing Points), 3000 iterations, and a latent space dimensionality capturing the underlying structure in the data (Q = 3).

### Iterations and Evolution

After establishing the baseline, experiments were conducted where parameters related to the number of iterations, inducing points, batch size, and latent space dimensionality were varied. In each case, the normalized mean squared error was reported as a combined metric for predicting newborn weight (regression) and predicting low birth weight (classification). Additionally, the $\:{R}^{2}$ and accuracy metrics were presented for each output scenario as independent measures for comparison with the single-output results.

### Tools

All tests were conducted using Google Colaboratory, an open-source project based on Jupyter that provides a virtual machine for executing Python code in version 3.7.13. The following libraries were used for the implementation of HETMOG: GPy, a toolbox for Gaussian processes; Climin, an optimization toolbox; and the implementations from Moreno et al. (2018) hetmogp and likelihoods hetmogp and likelihoods for constructing the multi-output model, from Giraldo et al. (2022) used convhetmogp_vik_fullcov for constructing the multi-output model. For data treatment and visualization, the tools used included Pandas, Numpy, Matplotlib, and Seaborn.

### General Approaches for Single-Output Models

Conventional single-output ML techniques, both applied to real and binary tasks, such as Linear Regression, Logistic Regression, Decision Trees, and SVM were used. All variables were included in the regression and classification models after preprocessing.

As an additional evaluation of the practical relationship between continuous and binary prediction, the outputs of the regression models were also translated into induced LBW classifications by applying the conventional 2,500 g threshold to the predicted birth weight values. Under this rule, births with predicted weight < 2,500 g were classified as LBW, whereas those with predicted weight ≥ 2,500 g were classified as non-LBW. Classification accuracy was then calculated for these induced classifications to assess the extent to which continuous birth-weight prediction could recover the binary LBW categorization. Further details on comparator single-output models, baseline settings, and the secondary induced-classification analysis are provided in the Supplementary Methods (Section S3).

### Baseline

Initial experiments involved evaluating metrics after conventional training and testing (70/30), using most default parameters of each method and all predictor variables. Subsequently, the parameters were fine-tuned via a grid search process while monitoring the metrics improvement. If the selected methods did not depend on parameters, such as in Linear or Logistic Regression, data treatment, output variable transformations, or dimensionality reduction using PCA were applied. A fuller description of baseline specifications, tuning, and validation procedures is available in the Supplementary Methods (Section S4).

### Iterations and Evolution

In the cross-validation iterations, all data was used to randomly create 10 folds, where each fold constitutes a random split of training and validation data. In each iteration or fold, metrics were reported, and the average and standard deviation of the 10 folds constitute the comparison baseline for the multi-output models. Additional details on the cross-validation procedure are provided in the Supplementary Methods (Section S4).

### Tools

For this phase, Anaconda and Jupyter were used. Anaconda is an open-source distribution for package management and environment creation, facilitating the setup and reproducibility of the experiments conducted. Jupyter is a web-based interactive environment that allows the creation of notebooks integrating code, visualizations, and explanatory text. This combination of Anaconda and Jupyter provides a robust and efficient platform for data exploration, model implementation, and clear and organized presentation of results. The following libraries were used for model implementation: Pandas, Numpy, and Scikit-learn, with Matplotlib and Seaborn for visualization.

## Results

By means of a relevance analysis 8 variables were selected to train and evaluate the HETMOGP model: Gestational age, mother’s age, baby’s sex, pregnancy multiplicity, number of prenatal consultations, type of delivery, month of birth, mother’s social security regime. These variables achieved an accumulated relevance of over 80%.

**Fig. 1:**
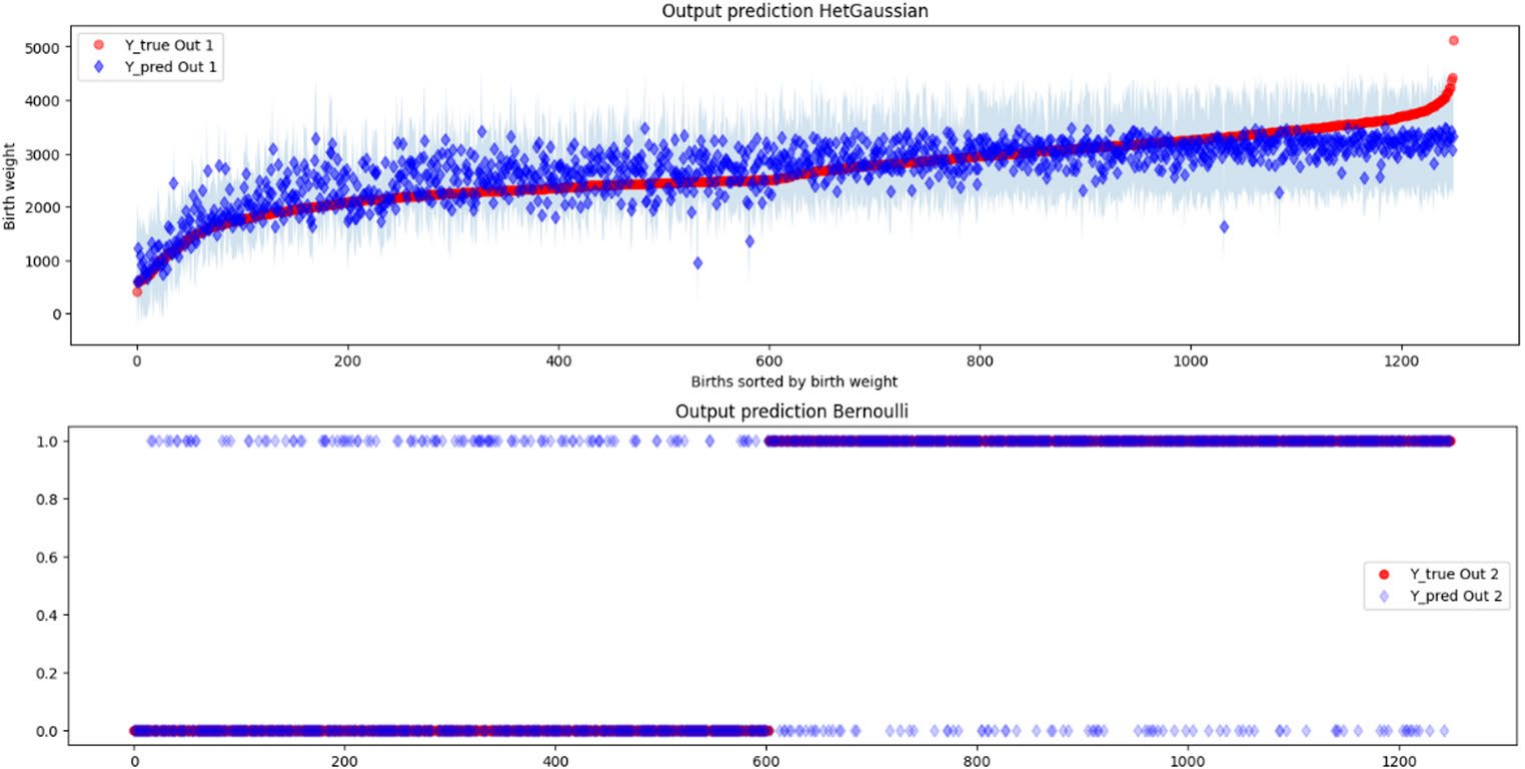
Prediction of newborn weight and low birth weight classification according to the HETMOGP model. In the upper panel, the red circles represent the observed newborn weights and the blue diamonds represent the predicted values for the regression task. In the lower panel, the red circles represent the observed low birth weight classification and the blue diamonds represent the predicted values for the classification task. HETMOGP = Heterogeneous Multi-Output Gaussian Process; Y_true = observed values; Y_pred = predicted values; LBW = low birth weight. In the upper panel, Out 1 denotes birth weight in grams. In the lower panel, Out 2 denotes LBW classification, coded as 1 for LBW and 0 for non-LBW. The shaded region in the upper panel represents the predictive uncertainty interval around the estimated birth weight trajectory

**Fig. 2:**
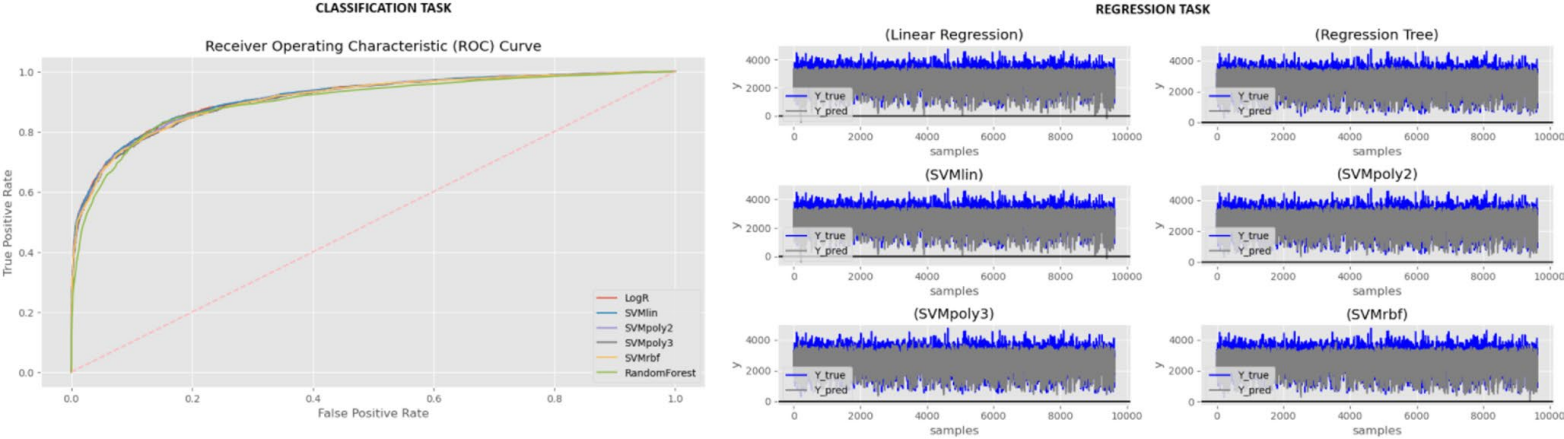
ROC curves for low birth weight classification models and observed versus predicted newborn weight for regression models. The left panel shows the receiver operating characteristic (ROC) curves for the independent classification task. The right panels show the observed newborn weights (blue) and the corresponding model estimates (gray) for the regression task. ROC = receiver operating characteristic; LogR = logistic regression; SVMlin = support vector machine with linear kernel; SVMpoly2 = support vector machine with second-degree polynomial kernel; SVMpoly3 = support vector machine with third-degree polynomial kernel; SVMrbf = support vector machine with radial basis function kernel; RandomForest = random forest; Y_true = observed values; Y_pred = predicted values

The experiments conducted to train and validate the HETMOGP model show stability in performance as the model parameters are varied. In all scenarios, MSE and Accuracy levels exceed 80%, and the variance explained in newborn weight is greater than 66%. The best performance is achieved with the parameters selected in Experiment 4. Figure 1 presents the model’s predictions for the two output variables: newborn weight and low birth weight classification. It shows that the weights predicted by the model (blue diamonds in Fig. 1) closely match the actual newborn weight values (red points in Fig. 1). It is noteworthy, as in single-output models, that prediction accuracy decreases when the newborn weight is high (see Fig. 2). Additionally, Fig. 1 demonstrates that when contrasting the prediction of low-birth-weight classification, there are relatively few blue diamonds or predicted values that do not align with the actual classification or red points in Fig. 1.

For the single-output ML classification task approaches (see Table 2), it is evident that the methods with the highest R² and lowest MAE are Linear Regression and Support Vector Machines (SVM) with a second-degree polynomial kernel. However, Decision Trees and other SVM kernels provide results very close to the best-performing methods. After cross-validation, there is little variability from one-fold to another, validating the initial results of the methods. It can be stated that the average performance of R² and MAE is 0.6748 ± 0.0076 and 299.7097 ± 3.8662 for SVM with a second-degree polynomial kernel, and 0.6723 ± 0.007 and 303.14791 ± 3.7095 for Linear Regression. This means that the selected methods are capable of explaining 67% of the variability in newborn weight with an estimation error close to 300 g.

**Table 2:** Variation of Parameters and Performance of Different HETMOGP and Machine Learning Models for Newborn Weight Prediction and Low Birth Weight Classification

HETMOGP														
	Variables	Experimentos												
		1	2	3		4		5		6		7		8
Model parameters	Q	2	2	2		3		5		5		5		5
	Optimizer	Adam	Adam	Adam		Adam		Adam		vo		vo		vo
	Minibatch	250	250	250		250		250		250		250		250
	Induced points	50	150	25		25		25		25		25		25
	Iterations	3000	3000	3000		3000		3000		2000		3000		5000
Performance	Standardized MSE	0.834	0.834	0.838		0.815		0.811		0.823		0.823		0.821
	R^2^	0.663	0.652	0.657		0.671		0.677		0.667		0.666		0.661
	Accuracy	0.843	0.842	0.836		0.845		0.842		0.843		0.844		0.844

Machine Learning					
Regression			Classification		
Method	R²	MAE	Method	Accuracy	AUC
Linear Regression	0.6720	300.89	LogR	0.8362	0.8367
Regression Tree	0.6423	307.56	SVMlin	0.8339	0.8344
SVMlin	0.6703	301.01	SVMpoly2	0.8310	0.8317
SVMpoly2	0.6718	299.13	SVMpoly3	0.8267	0.8273
SVMpoly3	0.6604	304.41	SVMrbf	0.8278	0.8287
SVMrbf	0.6659	302.57	RandForest	0.8371	0.8373

*HETMOGP* Heterogeneous Multi-Output Gaussian Process, *Q* number of latent processes, *Adam* Adaptive Moment Estimation, *vo* variational optimization, *MSE* mean squared error, *R*² coefficient of determination, *MAE* mean absolute error, *LogR* logistic regression, *SVMlin* support vector machine with linear kernel, *SVMpoly2* support vector machine with second-degree polynomial kernel, *SVMpoly3* support vector machine with third-degree polynomial kernel, *SVMrbf* support vector machine with radial basis function kernel, *RandForest* random forest, *AUC* area under the receiver operating characteristic curve

To further examine the practical relationship between continuous birth-weight prediction and the binary LBW definition, we applied the conventional 2,500 g cutoff to the predicted values generated by the regression models. Under this induced classification rule, accuracy ranged from 0.7754 for linear regression to 0.8122 for the regression tree model, indicating that the continuous-output models recovered a substantial proportion of the binary LBW classification despite not being trained directly for that task. Detailed model-specific results for this secondary analysis are reported in the Supplementary Methods (Section S3, Section S6, and Table S3).

For classification models, Random Forests provide the highest accuracy and area under the curve (AUC), followed by linear SVM and Logistic Regression. In other techniques, ROC curves are very close, with AUCs exceeding 0.80 in all cases. This is a strong sign of the models’ ability to accurately distinguish between infants classified as low birth weight and those not classified as such, suggesting a low rate of false positives and false negatives in classification. In cross-validation, for predicting low birth weight, the selected methods offer more stable metrics in each fold, with average accuracy and AUC of 0.8349 ± 0.0105 and 0.8933 ± 0.0088 for Random Forest, and 0.8310 ± 0.0112 and 0.8933 ± 0.0075 for Logistic Regression. The low variability in cross-validation confirms that the selected methods have an accuracy greater than 80% in predicting low birth weight classification.

## Discussion

In the searches conducted, no previous applications of multi-output models for predicting low birth weight were identified. However, the results for predicting birth weight using single-output methods were similar to those reported by Martínez et al. (2021), who used fetal ultrasound variables to train a single-output Gaussian process model with an error close to 300 g. In contrast, the classification results obtained in this study were better than those reported by Cobas et al. (2020), who used logistic regression with clinical and demographic variables to predict low birth weight and reported an ROC value of 0.759. They were also similar to the findings of Rigo et al. (2021), who used logistic regression to examine variables related to maternal characteristics and prenatal care, reporting an ROC value of 0.81.

These findings support the usefulness of information recorded in live birth certificates for predicting low birth weight in this specific context, as the predictive performance was comparable to that reported for models based on other types of data. In addition, the HETMOGP model achieved predictive performance similar to that of two separately fitted models for two related tasks within a single probabilistic framework.

An important point in interpreting these findings is that the two modeled outputs are not clinically independent. Low birth weight is a threshold-based categorization derived directly from birth weight in grams; therefore, this application should not be interpreted as jointly modeling two distinct perinatal outcomes that provide separate clinical information. Rather, its value lies in showing that an HETMOGP framework can accommodate heterogeneous outputs of different statistical types within a unified probabilistic model while achieving predictive performance comparable to that of separately fitted models.

In addition, in practical settings, an investigator interested in both birth weight in grams and low birth weight classification might reasonably fit a single model for the continuous outcome and then derive the binary classification using the conventional threshold, rather than fitting two entirely separate models. In our data, this indirect strategy retained reasonably good classification accuracy when applied to the regression predictions, suggesting that clinically relevant threshold-based categorization may be partially recovered from the continuous birth-weight estimates. The present study was not intended to argue against that strategy or to establish a demonstrated applied advantage over it. Instead, it was designed to examine the feasibility of a heterogeneous multi-output framework in which both representations are modeled jointly within a single probabilistic structure.

From a public health perspective, these findings suggest that routinely collected perinatal information may support the anticipation of low birth weight in this setting. Kristiawati et al. (2023), reported that parents of low-birth-weight newborns often experience uncertainty at discharge and require guidance regarding routine care. Although the present study should be interpreted primarily as a methodological demonstration, earlier identification of newborns at risk of low birth weight could, in principle, contribute to better preparation of parents and caregivers, as well as improved follow-up, care planning, and support after birth.

### Interpretation of the Multi-output Formulation in Derived Outcomes

An important consideration in this study is that the two modeled outputs, birth weight in grams and its low birth weight (LBW) classification, are not statistically independent. The binary LBW variable is a deterministic threshold-based transformation of the continuous birth weight outcome. As a result, the joint modeling framework does not introduce additional information in the sense typically assumed in multi-task learning settings, where multiple outputs provide complementary or partially independent signals.

In this context, the purpose of the multi-output formulation should be interpreted differently from conventional multi-task learning applications. Specifically, the present study does not aim to demonstrate performance gains from cross-output information sharing, nor to suggest that the binary outcome provides novel predictive information beyond the continuous measure. Instead, this setup provides a controlled setting in which the behavior of a heterogeneous multi-output Gaussian process model can be examined when combining likelihoods of different statistical types within a unified probabilistic framework.

From a methodological perspective, this formulation provides a useful test of the model’s ability to coherently represent and jointly infer a continuous outcome (via a Gaussian likelihood) and a derived categorical outcome (via a Bernoulli likelihood) through shared latent functions. This allows the assessment of model stability, convergence behavior, and predictive consistency under heterogeneous likelihood coupling, without the confounding influence of independently varying outputs.

Accordingly, this study should not be interpreted as evidence of the practical superiority of multi-output modeling over simpler approaches, such as fitting a regression model for birth weight and deriving LBW classification through thresholding. Rather, it should be understood as a proof-of-concept demonstrating the feasibility and internal consistency of a heterogeneous probabilistic framework when applied to real-world perinatal data.

Future research is needed to evaluate the full potential of this approach in settings involving multiple clinically distinct but correlated outcomes, where joint modeling may yield substantive gains in predictive performance and clinical utility.

## Conclusion

Traditional single-output models showed good predictive performance for both birth weight estimation and low birth weight classification. These findings support the practical usefulness of information recorded in live birth certificates for predicting birth weight and low birth weight classification in this setting and may help inform differentiated care strategies during pregnancy and after birth.

The results also position the HETMOGP model as a viable and flexible methodological alternative for jointly modeling birth weight and its low-birth-weight classification in this dataset. In this application, its performance was comparable to that of conventional models fitted separately for regression and classification. Because low birth weight is derived directly from birth weight, these findings should not be interpreted as evidence that joint modeling of the two outputs provides additional clinical information. Future studies may better demonstrate the applied value of this approach by considering multiple correlated outcomes that are clinically distinct.

## Supplementary Information

Below is the link to the electronic supplementary material.

Supplementary Material 1

## Data Availability

The data is available from an official Colombian open data source see https://medata.gov.co/dataset/1-026-22-000029.

## Code Availability

The codes used are available upon request from the authors.
