# Supplementary material for: Multiple Output Gaussian Process Model for Predicting Low Birth Weight in Medellín, Colombia: An Alternative to Conventional Machine Learning Models
Source: Matern Child Health J. 2026 Jun 29;30(7):875–83. doi: 10.1007/s10995-026-04284-x (PMC13451375; doi:10.1007/s10995-026-04284-x)
Supplement: Supplementary file 1 [file 10995_2026_4284_MOESM1_ESM.docx]

**SUPPLEMENTARY METHODS**

**Supplementary Methods for Data Preprocessing, Comparator Models, and HETMOGP Implementation**

**Purpose.** This supplementary document provides additional methodological detail on data preprocessing, comparator single-output models, tuning and validation procedures, the HETMOGP implementation, performance metrics, and software environment. It is intended to complement the main Methods section and improve transparency and reproducibility.

**S1. Data source and analytic dataset**

The original data were obtained from MeData, the institutional open-data repository of the Municipality of Medellín, Colombia. The source file contained records of live births occurring between 2012 and 2021. The original dataset included 272,526 records and 40 variables related to birth characteristics, maternal characteristics, prenatal care, and residence information.

After an initial screening based on clinical relevance and usefulness for predictive modeling, the dataset was cleaned by removing duplicate, incomplete, and inconsistent records. This yielded an intermediate dataset with 146,801 births and 20 candidate predictors. Because only 10.9% of these births (16,055 records) were classified as low birth weight (LBW), a random down-sampling strategy was applied to the non-LBW class to obtain a balanced analytic dataset with 32,110 records. The final analytic dataset comprised 20 predictors and 2 study outcomes (22 variables in total).

The two study outcomes were: (1) birth weight in grams (continuous outcome; PESO_NAC) and (2) LBW status (binary outcome; BAJO_PESO), defined using the conventional threshold of less than 2,500 g. All remaining variables were treated as candidate predictors.

**Table S1. Variables included in the analytic dataset.**

| **Variable** | **Description** |
| --- | --- |
| AREANAC | Area of birth |
| SIT_PARTO | Place of delivery |
| SEXO | Sex of the newborn |
| MES | Month of occurrence |
| ATEN_PAR | Birth attendant |
| T_GES | Gestational age/time of gestation |
| NUMCONSUL | Number of prenatal visits |
| TIPO_PARTO | Type of delivery |
| MUL_PARTO | Multiplicity of pregnancy |
| IDHEMOCLAS | Newborn blood group |
| IDFACTORRH | Newborn Rh factor |
| EDAD_MADRE | Maternal age at delivery |
| EST_CIVM | Maternal marital status |
| NIV_EDUM | Maternal education |
| N_HIJOSV | Number of previous live births |
| N_EMB | Number of pregnancies, including the current pregnancy |
| SEG_SOCIAL | Maternal health insurance regime |
| EDAD_PADRE | Paternal age at birth |
| NIV_EDUP | Paternal education |
| COMUNA_RES | Maternal commune of residence |
| PESO_NAC | Birth weight at delivery (g) |
| BAJO_PESO | Low birth weight status (< 2,500 g) |

The study used retrospectively collected, anonymized secondary data from the MeData repository. No direct personal identifiers were available in the analytic dataset.

**S2. Data preprocessing**

Data preprocessing involved four main stages: data cleaning, variable selection, class balancing, and feature transformation.

**Cleaning:** Duplicate records, incomplete observations, and inconsistent or implausible values were removed before model fitting.

**Variable selection:** Predictors were retained according to clinical relevance and the possibility of being measured before birth, so that the resulting models would preserve a meaningful predictive interpretation.

**Class balancing:** Because LBW was the minority class, a random sample of equal size was drawn from the non-LBW class to create a balanced dataset for model training and comparison.

**Encoding and scaling:** For conventional single-output models, categorical variables were transformed using one-hot encoding. For the HETMOGP model, categorical variables were label-encoded to avoid an excessive increase in dimensionality during optimization. Numerical predictors were rescaled using MinMaxScaler to place variables on comparable scales before training.

The same preprocessed analytic dataset was used for the baseline comparison of the single-output models, whereas the HETMOGP implementation used label-encoded categorical variables to reduce dimensionality during optimization.

**S3. Comparator single-output models**

The performance of the HETMOGP model was compared against conventional single-output methods fitted separately for the two study tasks: regression of birth weight in grams and binary classification of LBW status.

For the regression task, the comparator models reported in the main manuscript were linear regression, regression trees, and support vector machines (SVMs) with linear, polynomial, and radial basis function kernels.

For the classification task, the comparator models reported in the main manuscript were logistic regression, support vector machines (SVMs) with linear, polynomial, and radial basis function kernels, and random forest.

All comparator models were initially fitted using the full set of predictors after preprocessing. The baseline comparison was based on a conventional 70/30 split into training and validation subsets. The best-performing models from this initial comparison were then selected for additional tuning and cross-validation.

**Table S2. Comparator single-output models and baseline settings.**

| **Task** | **Method** | **Baseline specification** |
| --- | --- | --- |
| Regression | Linear regression | Default implementation with all predictors after preprocessing. |
| Regression | Regression tree | Default decision-tree regression implementation. |
| Regression | SVM (linear, polynomial, RBF) | Support vector regression models with kernel-specific baseline settings. |
| Classification | Logistic regression | Default implementation with all predictors after preprocessing. |
| Classification | SVM (linear, polynomial, RBF) | Support vector classification models with kernel-specific baseline settings. |
| Classification | Random forest | Default random-forest classifier. |

For the secondary evaluation of regression outputs as induced LBW classifiers, the conventional 2,500 g threshold was applied to the predicted birth-weight values. Under this rule, predicted birth weight < 2,500 g was classified as LBW and predicted birth weight ≥ 2,500 g as non-LBW. Detailed model-specific results for this secondary analysis are reported in Table S3.

**Table S3. Classification accuracy obtained after applying the 2,500 g threshold to the birth-weight predictions from the regression models**

|  | Induced LBW classification | | |
| --- | --- | --- | --- |
|  | Method |  | Accuracy |
|  | Linear Regression | | 0.7754 |
|  | Regression Tree |  | 0.8122 |
|  | SVMlin |  | 0.7768 |
|  | SVMpoly2 |  | 0.7851 |
|  | SVMpoly3 |  | 0.7895 |
|  | SVMrbf |  | 0.7843 |

***Abbreviation****s: LBW, low birth weight; SVMlin, support vector machine with linear kernel; SVMpoly2, support vector machine with second-degree polynomial kernel; SVMpoly3, support vector machine with third-degree polynomial kernel; SVMrbf, support vector machine with radial basis function kernel.*

***Note:*** *LBW classification was induced by applying the conventional 2,500 g threshold to the continuous birth-weight predictions generated by each regression model.*

**S4. Tuning and validation strategy**

The initial baseline comparison used a random 70/30 split for training and validation. After this baseline stage, the two best-performing models for each task were selected for additional optimization. When the selected methods involved tunable hyperparameters, parameter values were refined using grid search over prespecified candidate values. For models with little or no meaningful hyperparameter dependence in this context, additional preprocessing strategies such as output transformation or principal component analysis were considered when appropriate.

The tuned single-output models were then evaluated by 10-fold cross-validation on the balanced analytic dataset used for model comparison. In each fold, model performance was estimated on the held-out partition and the final summary was reported as the mean and standard deviation across the 10 folds. These cross-validated results were used as the main baseline for comparison with the multi-output HETMOGP approach.

**S5. HETMOGP implementation**

Because of the computational burden associated with HETMOGP optimization, feature selection was performed before training the multi-output model. Feature importance was estimated using a Light Gradient Boosting Machine (LightGBM), and the final subset retained those variables whose cumulative importance reached at least 80%.

The HETMOGP model was specified with two heterogeneous outputs: a Gaussian likelihood for birth weight in grams and a Bernoulli likelihood for LBW status. The implementation followed the extension of the HETMOGP framework described by Giraldo et al. (2022), in which heterogeneous outputs are handled through a correlated chained Gaussian-process formulation.

The initial HETMOGP baseline used the Adam optimizer because of its favorable convergence behavior in preliminary experiments. The starting configuration consisted of a minibatch size of 250, 50 inducing points, 3,000 iterations, and a latent dimensionality of Q = 3. After establishing this baseline, additional experiments were conducted by varying the number of iterations, the number of inducing points, minibatch size, latent dimensionality, and optimizer. Performance was tracked jointly through standardized mean squared error and separately through regression and classification metrics for each output.

**S6. Performance metrics**

For the regression task (birth weight prediction), performance was summarized using mean absolute error (MAE) and the coefficient of determination (R²). For the classification task (LBW prediction), performance was summarized using accuracy and the area under the receiver operating characteristic curve (AUC).

For the secondary evaluation of regression outputs as induced LBW classifiers, performance was summarized using classification accuracy after applying the conventional 2,500 g threshold to the predicted birth-weight values. Detailed results are reported in Table S3.

For the HETMOGP model, a standardized mean squared error was additionally reported as a combined summary of performance across the continuous and binary outputs, while task-specific metrics were retained to enable direct comparison with the corresponding single-output models.

**S7. Software environment**

The single-output comparator models were implemented in Python using the Anaconda distribution and Jupyter notebooks. Data manipulation and modeling relied primarily on pandas, NumPy, and scikit-learn, with matplotlib and seaborn used for visualization.

The HETMOGP experiments were run in Google Colaboratory using Python 3.7.13. The Gaussian-process implementation relied on GPy, climin, the hetmogp and likelihoods toolboxes associated with Moreno-Muñoz et al. (2018), and the convhetmogp_vik_fullcov implementation described by Giraldo et al. (2022).

**References cited in the supplementary methods**

Giraldo, J. J., Zhang, J., & Álvarez, M. A. (2022). Correlated Chained Gaussian Processes for Modelling Citizens Mobility Using a Zero-Inflated Poisson Likelihood. IEEE Transactions on Intelligent Transportation Systems, 23(11), 20337–20351.

Ke, G., Meng, Q., Finley, T., Wang, T., Chen, W., Ma, W., Ye, Q., & Liu, T.-Y. (2017). LightGBM: A Highly Efficient Gradient Boosting Decision Tree.

Liu, H., Cai, J., & Ong, Y. S. (2018). Remarks on multi-output Gaussian process regression. Knowledge-Based Systems, 144, 102–121.

Moreno-Muñoz, P., Artés-Rodríguez, A., & Álvarez, M. A. (2018). Heterogeneous Multi-output Gaussian Process Prediction.

Rasmussen, C. E., & Williams, C. K. I. (2006). Gaussian Processes for Machine Learning. MIT Press.

Xu, D., Shi, Y., Tsang, I. W., Ong, Y. S., Gong, C., & Shen, X. (2020). Survey on Multi-Output Learning. IEEE Transactions on Neural Networks and Learning Systems, 31(7), 2409–2429.
